# Identification of a prototype human gut *Bifidobacterium longum* subsp. *longum* strain based on comparative and functional genomic approaches

**DOI:** 10.3389/fmicb.2023.1130592

**Published:** 2023-02-08

**Authors:** Giulia Alessandri, Federico Fontana, Chiara Tarracchini, Sonia Mirjam Rizzo, Massimiliano G. Bianchi, Giuseppe Taurino, Martina Chiu, Gabriele Andrea Lugli, Leonardo Mancabelli, Chiara Argentini, Giulia Longhi, Rosaria Anzalone, Alice Viappiani, Christian Milani, Francesca Turroni, Ovidio Bussolati, Douwe van Sinderen, Marco Ventura

**Affiliations:** ^1^Laboratory of Probiogenomics, Department of Chemistry, Life Sciences, and Environmental Sustainability, University of Parma, Parma, Italy; ^2^GenProbio srl, Parma, Italy; ^3^Department of Medicine and Surgery, University of Parma, Parma, Italy; ^4^Microbiome Research Hub, University of Parma, Parma, Italy; ^5^APC Microbiome Institute and School of Microbiology, Bioscience Institute, National University of Ireland, Cork, Ireland

**Keywords:** probiotics, bifidobacteria, host–microbe interactions, microbe-microbe interactions, RNA sequencing, intestinal barrier, inflammation

## Abstract

Bifidobacteria are extensively exploited for the formulation of probiotic food supplements due to their claimed ability to exert health-beneficial effects upon their host. However, most commercialized probiotics are tested and selected for their safety features rather than for their effective abilities to interact with the host and/or other intestinal microbial players. In this study, we applied an ecological and phylogenomic-driven selection to identify novel *B. longum* subsp. *longum* strains with a presumed high fitness in the human gut. Such analyses allowed the identification of a prototype microorganism to investigate the genetic traits encompassed by the autochthonous bifidobacterial human gut communities. *B. longum* subsp. *longum* PRL2022 was selected due to its close genomic relationship with the calculated model representative of the adult human-gut associated *B. longum* subsp. *longum* taxon. The interactomic features of PRL2022 with the human host as well as with key representative intestinal microbial members were assayed using *in vitro* models, revealing how this bifidobacterial gut strain is able to establish extensive cross-talk with both the host and other microbial residents of the human intestine.

## Introduction

Members of the genus *Bifidobacterium* are abundant symbiotic microorganisms inhabiting the gastrointestinal tract of mammals (Alessandri et al., 2021). This bacterial genus currently counts more than 100 species (Lugli et al., 2021; Li et al., 2022), and in recent decades particular scientific focus has been addressed on *Bifidobacterium longum* subsp. *longum*, due to its purported health benefits and because it is the most prevalent and abundant bifidobacterial species colonizing the human intestine throughout the entire lifespan (Santos et al., 2021; Kim et al., 2022; Quintanilha et al., 2022). In this context, convincing scientific evidence has accumulated demonstrating that several *B. longum* subsp. *longum* strains are able to support intestinal barrier integrity, provide protection against pathogen proliferation, limit the onset/exacerbation of inflammatory gut diseases and metabolic disorders, and modulate host immunity to promote gut homeostasis (Schiavi et al., 2016, 2018; McCarville et al., 2017; Alessandri et al., 2019; Yan et al., 2019; Hao et al., 2022; Quintanilha et al., 2022). Furthermore, *B. longum* subsp. *longum* strains not only significantly contribute to host metabolism by degrading, through saccharolytic activities, a wide variety of otherwise non-digestible glycans, but also produce various metabolites, including polyphenols, vitamins, conjugated linoleic acids and short-chain fatty acids (SCFAs) that are believed to beneficially impact on both the gut microbial ecosystem and epithelial host cells (Tanno et al., 2019; Blanco et al., 2020; Alessandri et al., 2021; Sasaki et al., 2021; Komeno et al., 2022).

However, despite clear experimental evidence supporting the purported probiotic features of certain *B. longum* subsp. *longum* strains, the molecular mechanisms behind such health-promoting activities are still far from being fully understood (Turroni et al., 2014; Hidalgo-Cantabrana et al., 2017; Kim et al., 2018). Certainly, the rapid evolution of systems biology toward high-throughput “omics” technologies allowed mechanistic investigation of probiotic actions, an approach sometimes also referred to as probiogenomics (Ventura et al., 2009, 2012; Turroni et al., 2014). The latter has, in particular due to genome decoding and functional analysis of bacterial strains, contributed to improve insights into the diversity and evolution of health-promoting bacteria, while in some cases it has also shed light on the mechanisms underlying their beneficial effects (Ventura et al., 2012; Turroni et al., 2018a; Choi et al., 2021). However, despite our deepening insights into probiotic mechanisms, most of the probiotic strains that are currently on the market have been tested and selected principally for their survival and safety features, thus limiting knowledge about the ability of these strains to interact with their host and/or with other microbial players of the human gut environment.

In this context, to identify a prototype human gut *B. longum* subsp. *longum* strain with a presumed high fitness in the human gut, we applied an ecological and phylogenomic-driven selection approach. These analyses allowed identification of *B. longum* susp. *Longum* PRL2022 as the genetically and functionally closest strain to the most representative strains of this bifidobacterial species present in the human intestinal microbiota, thus highlighting this microorganism as a model strain for this (sub)species. Genomic insights of PRL2022 were obtained, and a comparative genomic analysis was performed that included the genome of PRL2022 and those of *B. longum* subsp. *longum* strains that are currently included in commercially available probiotic products, leading to the identification of PRL2022-associated unique genes putatively involved in host- and/or microbe-microbe interactions, suggesting its adaptation to the human gut ecological niche. In order to validate such *in silico*-based data, functional genomic experiments using *in vitro* models of the human gut were carried out, showing how this putative model microorganism is able to establish extensive cross-talks with both the host and other microorganisms of the intestinal ecosystem.

## Materials and methods

### Dataset selection

All publicly available datasets corresponding to fecal samples of healthy adults sequenced through a shotgun metagenomics approach were selected and downloaded from the National Center for Biotechnology Information (NCBI) SRA repository using NCBI SRA Toolkit 2.11.0 faster-dump (Leinonen et al., 2011). Specifically, to achieve high quality and coverage data, only shotgun metagenomic data sets based on Illumina sequencing technology were retained (Supplementary Table S1).

### *Bifidobacterium longum* subsp. *longum* genome selection

To select a model candidate of the *B. longum* subsp. *longum* species, all publicly available *B. longum* subsp. *longum* genomes from the NCBI genome list were downloaded and then subjected to genome quality assessment. In detail, only genome sequences with a genome coverage higher than 30-fold and containing less than 100 contigs were considered. Subsequently, the retained high-quality genomes were processed through the DRep and CheckM tools to cluster identical genomes, i.e., those with an Average Nucleotide Identity (ANI) ≥ 99%, to eliminate redundant genomes, and to obtain a high-quality non-redundant *B. longum* subsp. *longum* genome database (Supplementary Table S2).

### Identification of novel model candidates among the *Bifidobacterium longum* subsp. *longum* genomic database

The retained high-quality non-redundant *B. longum* subsp. *longum* genomes together with the.fastq corresponding to the selected shotgun metagenomics sequencing-based publicly available dataset from adult fecal samples were submitted to and processed by the open-source Strain Genome Explorer (StrainGE) software^1^ (Supplementary Tables S1, S2). Specifically, Strain Genome Search Tool (StrainGST), i.e., an integrated tool of the StrainGE suite, allowed detection of bifidobacterial strains included in the abovementioned *B. longum* subsp. *longum* genomic database in the selected shotgun metagenomics dataset of fecal samples from adult (van Dijk et al., 2022). StrainGE allows assessment of the prevalence of each genome contained in the *B. longum* subsp. *longum* genomic database within the analyzed shotgun metagenomics samples. Subsequently, to identify the model candidate among the *B. longum* subsp. *longum* strains included in the genomic database, we assessed the AxP index, defined as [average ANI value calculated for each genome] * [prevalence score of the strain in the shotgun metagenomics dataset] * [100] (Fontana et al., 2022).

### Application of the RefBifSelector

Since the identified optimal candidate among the *B. longum* subsp. *longum* genomic database is not available in our laboratory, to identify, among those strains available in our local bacterial repository, a *B. longum* subsp. *longum* model strain genomically and functionally closest to the above-identified optimal candidate, the RefBifSelector tool was used (Fontana et al., 2022). In detail, the tool enables the identification of the strain, within a local biobank repository, that is genomically the most closely related to the identified optimal model strain, as based on a genomic comparison. Specifically, this selection is driven by ANI value in conjunction with the use of the average Percentage of Positive Scoring matches (PPOS). While ANI analysis investigates the nucleotide identity between genome pairs, the PPOS score is calculated from the comparison of the translated amino acid sequences obtained through Blastp analysis following the [(number of identical matches) + (number of similar matches)]/(alignment length) formula (Fontana et al., 2022).

### Genome sequencing and assembly

To obtain a fully representative sequence of *B. longum* subsp. *longum* PRL2022 genome, the previously obtained sequencing data from Illumina platform were combined with long reads obtained from genome sequencing on a MinION sequencer (Oxford Nanopore, United Kingdom). Specifically, the DNA extracted from the selected strain was subjected to whole-genome sequencing using a MinION (Oxford Nanopore, United Kingdom). DNA library preparation was performed using the Native Barcoding Genomic DNA kit according to the manufacturer’s instructions. One microgram input DNA from each sample was used for library preparation. The samples were pooled and diluted to a final concentration of 5–50 fmol. Sequencing was performed on MinIon device (Oxford Nanopore, United Kingdom) using a R9.4.1 flow cell. Long reads were filtered by quality using the Filtlong tool,^2^ while short reads were filtered through the fastq-mcf script.^3^ Then, filtered fastq files of MinION long reads obtained from genome sequencing efforts were used as input for genome assembly through CANU software (Koren et al., 2017). The resulting genome sequences have been polished through Polypolish (Wick and Holt, 2022) using Illumina paired-end reads (250 bp). Polished contigs were then employed by MEGAnnotator (Lugli et al., 2016) for the prediction of protein-encoding open reading frames (ORFs) using Prodigal (Hyatt et al., 2010).

### Gene annotation and prediction of genes involved in host- and microbe-microbe interaction in PRL2022 genome

Open reading frames (ORFs) in the PRL2022 genome were predicted and annotated using the most recent release of the MEGAnnotator pipeline (Lugli et al., 2016). In detail, contigs longer than 1,000 bp were employed to predict protein encoding ORFs through Prodigal v2.0 (Hyatt et al., 2010). Predicted ORFs were then functionally annotated using RAPSearch2 (cutoff e-value of 1 × 10^−5^ and minimum alignment length 20) using the NCBI reference sequences (RefSeq) database (Zhao et al., 2012a) coupled with hidden Markov model profile (HMM) searches against the manually curated Pfam-A database (cutoff *e*-value of 1 × 10^−10^). The PRL2022 genome sequence was subjected to prediction of genes encoding glycosyl hydrolases (GHs), glycosyl transferases (GTs), carbohydrate binding modules (CBMs), and carbohydrate esterases (CEs) through sequence similarity search in the carbohydrate-active enzyme (CAZy) database (Lombard et al., 2014) using HMMER v3.3 (Wheeler and Eddy, 2013) (cutoff *e*-value of 1 × 10^−15^) and BLASTP analysis (Altschul et al., 1990) (cutoff *e*-value of 1 × 10^−10^). The presence of pilus gene clusters was scrutinized through homology search tool RAPSearch2 (cutoff *e*-value of 1 × 10^−5^ and minimum alignment length 20) (Zhao et al., 2012a) exploiting the custom pilus gene database previously described (Milani et al., 2017). In addition, to identify loci encoding exopolysaccharides, protein sequences of priming glycosyltransferases (pGTFs) were retrieved from the NCBI database and were compared against the predicted PRL2022 proteome. Once the putative pGTF was identified, the genomic regions flanking the corresponding gene were investigated to identify genes involved in EPS biosynthesis, i.e., glycosyl transferases, flippases, ABC transporters, and carbohydrate precursor biosynthesis/modification enzymes. Finally, the presence of putative bile salt hydrolase- and serpin-encoding genes was investigated through sequence similarity searches in the protein sequence NCBI database (cutoff *e*-value of 1 × 10^−5^).

### Comparative genome analysis

The PRL2022 genome together with various genomes of *B. longum* subsp. *longum* strains that are currently included in a variety of commercially available probiotic products (Tarracchini et al., 2022; Supplementary Table S3), were subjected to a pangenome analysis pipeline (PGAP) (Zhao et al., 2012b). Predicted proteome of a specific *B. longum* subsp. *longum* strain was screened for orthologous enconding genes against the proteome of the other considered *B. longum* subsp. *longum* strains employing BLAST analysis (cutoff *e*-value of 1 × 10^−10^ and exhibiting at least 50% identity across at least 80% of both protein sequences) (Altschul et al., 1990). The obtained data were then clustered into protein families, i.e., clusters of orthologous genes (COGs) employing the Markov clustering algorithm (Enright et al., 2002), by means of the method gene family (GF). Based on the presence/absence matrix encompassing all COGs identified in the analyzed genomes, unique genes present in PRL2022 genome and not in the other 10 considered genomic sequences were identified. Functional annotation of each unique gene was accomplished using the Eggnog database (Huerta-Cepas et al., 2016).

### Cultivation conditions

PRL2022 strain was cultivated in the de Man-Rogosa-Sharpe (MRS) medium (Sharlau Chemie, Spain) supplemented with 0.05% (wt/vol) L-cysteine hydrochloride (Merk, Germany) and incubated at 37°C in a chamber (Concept 400, Ruskinn) with an anaerobic atmosphere (2.99% H_2_, 17.01% CO_2_, and 80% N_2_).

### pH, sodium chloride, and bile salts tolerance tests

To evaluate the ability of the selected strain to tolerate various pH levels, *B. longum* subsp. *longum* PRL2022 was cultivated in 10 mL of MRS broth at 37°C under anaerobic conditions to reach a final concentration of 10^8^ cells/mL. Subsequently, cells were centrifuged at 3,000 rpm for 8 min, washed with a saline solution (PBS, pH 6.5) and resuspended in 10 mL of MRS broth whose pH was adjusted to pH 2.0, pH 3.0, or pH 4.0 with the addition of HCl. Cells were incubated under anaerobic conditions at 37°C for 2 h, as previously described (Yasmin et al., 2020). The same procedure was performed to assess the ability of PRL2022 to tolerate different NaCl (2, 6, and 10%) or bile salt (0.5, 1, and 2%) concentrations with an exposure of 3 h to these stressful conditions, as previously reported (Yasmin et al., 2020). All experiments were carried out in triplicate and a control sample was obtained by inoculating PRL2022 cells in MRS broth. After incubation, cell viability was evaluated by means of the LIVE/DEAD BacLight Bacterial Viability kit (ThermoFisher Scientific, United States) and an Attune NxT flow cytometer (ThermoFisher Scientific, United States).

### Flow cytometry bacterial viability assay

Following exposure to acidic environment, or various bile salt or NaCl concentrations, a 10-fold serial dilution in Phosphate Buffered Saline (PBS) was obtained from each tested condition. The diluted cells were then used for a flow cytometry cell viability assay using the fluorescent dyes SYTO9 (3.34 mM) and PI (20 mM) of the LIVE/DEAD BacLight Bacterial Viability kit (ThermoFisher Scientific, United States), following the manufacturer’s protocol (Manual of the LIVE/DEAD BacLight Bacterial Viability and counting kit, ThermoFisher Scientific, United States). Briefly, two aliquots of 1 ml of bacterial cell dilution (1:1000) were harvested by centrifugation at 3,000 rpm for 8 min and washed with PBS. Subsequently, one of the two aliquots of bacterial suspension was exposed to 70% isopropyl alcohol and kept on ice for 1 h to permeabilize cell membranes and cause cell death, while the other 1 mL aliquot was maintained in PBS to preserve cell viability. Subsequently, 1.5 μL of a specific dye was added to samples for single staining assay, while 1.5 μL of both dyes were added for the double staining assay. Once stained, samples were incubated in the dark for 15 min at room temperature. Furthermore, while single-stained controls were used for instrument parameter adjustment, non-stained cells were used as a background control. Cell viability assay was performed with the Attune NxT flow cytometer (ThermoFisher Scientific, United States), and all data were analyzed with the Attune NxT flow cytometer software.

### Antibiotic susceptibility assay

Strain PRL2022 susceptibility to ampicillin, vancomycin, gentamicin, kanamycin, streptomycin, erythromycin, clindamycin, tetracycline, and chloramphenicol was investigated by Minimum Inhibitory Concentration (MIC) assays using the broth microdilution method (MDIL) following the European Food Safety Authority (EFSA) recommendations (Masco et al., 2006; EFSA, 2012). Briefly, from each antibiotic stock solution, two-fold dilution series were obtained and aliquoted in a 96-well microtiter plate. Subsequently, an overnight culture of strain PRL2022 was diluted to obtain an Optical Density at 600 nm (OD_600nm_) = 1, and 15 μL of the diluted cells were inoculated in 135 μL of culture medium supplemented with a specific antibiotic concentration. Microplates were incubated under anaerobic conditions at 37°C for 48 h. The MIC breakpoints expressed in μg/mL represent the highest concentration of a given antibiotic to which the strain was shown to be resistant. All experiments were performed in triplicate. The same procedure was used for the quality control strain *B. longum* subsp. *longum* ATCC 15707^T^ (ES ISO 10932:2010).

### Human cell line culture

Human colorectal adenocarcinoma-derived Caco-2 cells (purchased from ATCC) and human colon carcinoma-derived mucin-secreting goblet cells HT29-MTX (kindly provided by Prof. Antonietta Baldi, University of Milan) were cultured in Minimum Essential medium (MEM) and Dulbecco’s Modified Eagle’s medium (DMEM) with high glucose (4.5 g/L) and 10 mM of sodium pyruvate, respectively, as previously described (Bianchi et al., 2019). Both media were supplemented with 10% Fetal Bovine Serum (FBS), 2 mM glutamine, 100 g/mL streptomycin, and 100 U/mL penicillin. Cultures were maintained at 5% CO_2_ at 37°C and passaged three times a week. Subsequently, a mixed suspension of Caco-2 and HT29-MTX cells (7:3) was seeded in DMEM + FBS at a density of ≈10^5^ cells/cm^2^ into cell culture inserts with membrane filters (pore size 0.4 μm) for Falcon 24-well-multitrays (Becton, Dickinson & Company, Franklin Lakes, NJ, United States), and cultured for 21 days with a medium replacement every 3 days until a tight monolayer was formed (TEER >600 Ω • cm^2^).

### Human cell line monolayer in contact with PRL2022

After 21 days of seeding, the culture medium of the 24-well plates was replaced with fresh antibiotic-free DMEM. Subsequently, PRL2022 cells (final concentration 10^8^ cells/mL) were added to Caco-2/HT29-MTX cell monolayer, as previously described (Serafini et al., 2013; Fontana et al., 2022). The 24-well plates were subsequently incubated at 5% CO_2_ at 37°C. After 4 h of incubation, bacterial cells and human cell lines were separately recovered in RNA later and preserved at −80°C until processing.

Specifically, for this trial, *B. longum* subsp. *longum* PRL2022 was grown in MRS broth under anaerobic conditions at 37°C. Once the exponential growth phase (0.6 < OD_600nm_ < 0.8) was reached, bifidobacterial cells were enumerated by using the Thoma cell counting chamber (Herka), possibly diluted to reach a final concentration of 1 × 10^8^ cells/mL, washed in PBS, resuspended in 400 μL of antibiotic-free DMEM, and seeded on Caco-2/HT29-MTX cell monolayers. The strain PRL2022 resuspended in DMEM and maintained under the same incubation conditions of the 24-well plates without any contact with human cell lines was used as bacterial control sample, while Caco-2/HT29-MTX cell monolayers without any bifidobacterial seeding were used as human cell line control sample. All experiments were carried out in triplicate.

### Prokaryotic and eukaryotic RNA extraction

Total RNA from bacterial cells was isolated using a previously described method (Turroni et al., 2016; Milani et al., 2020). Briefly, bifidobacterial cell pellets were resuspended in 1 mL of QIAzol lysis reagent (Qiagen, Germany) in a sterile tube containing glass beads. Cells were lysed by alternating 2 min of stirring the mix on a bead beater with 2 min of static cooling on ice. These steps were repeated three times. Lysed cells were centrifuged at 12,000 rpm for 15 min, and the upper phase was recovered. Bacterial RNA was subsequently purified using the RNeasy Mini Kit (Qiagen, Germany) following the manufacturer’s instruction. Total RNA from human cell lines was extracted by adding 350 μL of RLT buffer from the RNeasy Mini Kit (Qiagen, Germany) and following the manufacturer’s instructions. In both cases, the RNA concentration and purity were evaluated using a spectrophotometer (Eppendorf, Germany).

### Prokaryotic and eukaryotic mRNA sequencing analysis

Total bacterial RNA (from 100 ng to 1 μg) was treated to remove rRNA by means of the QIAseq FastSelect – 5S/16S/23S following the manufacturer’s instructions (Qiagen, Germany). The yield of rRNA depletion was checked with a 2,200 TapeStation (Agilent Technologies, United States). Then, a whole transcriptome library for both prokaryotic and eukaryotic RNA was constructed using the TruSeq Stranded mRNA Sample preparation kit (Illumina, San Diego, USA). Samples were then loaded onto a NextSeq high-output v2 kit (150 cycles) (Illumina) as indicated by the technical support guide. The obtained prokaryotic reads were filtered to remove low-quality reads (minimum mean quality 20, minimum length 150 bp) as well as any remaining ribosomal locus-encompassing reads using the METAnnotator X2 pipeline (Milani et al., 2021). Subsequently, the retained reads were aligned to the specific reference genome through Bowtie2 software (Langdon, 2015), while high quality .fastq were aligned to the Human reference genome sequence (GRCh38.p13) by the use of the splice-aware STAR algorithm (version 2.7.10a) (Dobin et al., 2013), and quality of the alignments was evaluated using Picard software tool (version 2.26.11) (https://broadinstitute.github.io/picard/). Subsequently, quantification of reads mapped to individual transcripts was achieved through htseq-counts script of HTSeq software in “union” mode (Anders et al., 2015). Raw counts were then normalized using CPM (Counts per million mapped reads) for filtering genes with low counts (CPM <1) and TMM (Trimmed Mean of *M*-Values) for statistically robust differential gene expression analysis through the EdgeR package (Robinson et al., 2010). Evaluation of expression differences was calculated for each gene as log2 fold change (logFC) of average expression between the control (no contact between human cell lines and strain PRL2022) and “treated” samples (contact between human cell lines and strain PRL2022). Additionally, for each comparison, a Volcano plot was created to simultaneously visualize expression changes (log fold change) and their statistical significance (*value of p*).

### *In vitro* evaluation of PRL2022 response to human gut microbiota exposure

To evaluate how PRL2022 interacts with the gut microbial community, batch cultures were set up to co-cultivate the selected strain with two different bacterial communities, previously stabilized through a bioreactor system (Solaris Biotech Solutions, Italy) in IGS medium (Alessandri et al., 2022), dominated by species of either *Bacteroides* or *Prevotella*, i.e., two of the most abundant and representative genera of the human gut microbiota (Arumugam et al., 2011; Costea et al., 2018).

Batch cultures were obtained by inoculating 0.1% (vol/vol) of a stabilized intestinal microbial community and 1% (vol/vol) of an overnight culture of the strain PRL2022, as previously described (Mancabelli et al., 2021), in 30 mL of IGS medium adjusted to 6.8 ± 0.2 pH to mimicking the human intestinal environment (Alessandri et al., 2022). In addition, a batch culture with 1% (vol/vol) of strain PRL2022 was obtained as a control sample. All microbial cultures were performed in triplicate and incubated under anaerobic conditions at 37°C. After 8 h of incubation, cultures were centrifuged at 7,000 rpm for 5 min, the supernatants were discarded, while the obtained bacterial pellets were used for RNA extraction. RNA extraction and sequencing, as well as RNA sequencing analysis were performed as described bove.

### Statistical analysis

For differential gene expression analysis, EdgeR package was used to estimate the statistical significance of differences between fold changes as the False Discovery Rate (FDR).

### Data availability statement

Raw sequences of the RNAseq experiments are accessible under BioProject accession number PRJNA914637 and PRJNA833139. The updated genome sequence of *B. longum* subsp. *longum* PRL2022 is available under the accession number PRJNA692178.

## Results and discussion

### Ecological and phylogenomic-driven identification of model/prototype candidates belonging to the *Bifidobacterium longum* subsp. *longum* species

Bacterial strains that naturally colonize the human intestine are expected to possess high ecological fitness and to be equipped with a genomic assembly that supports their persistence in the intestinal environment. To identify a candidate prototype with a presumed high fitness in the human intestinal ecosystem among the *B. longum* subsp. *longum* strains whose genome sequences are publicly available, an ecological and phylogenomic-driven selection approach was performed, which is based on a recent published protocol with some modifications (Fontana et al., 2022). Specifically, all publicly available *B. longum* subsp. *longum* genomes were first checked for genome completeness, presence of contaminant genomic sequences and genome heterogeneity. Genomes that passed this initial quality check were then assessed for genome redundancy resulting in the retention of 306 high-quality, non-redundant *B. longum* subsp. *longum* genomes (Supplementary Table S2). Then, a strain tracking analysis was performed to predict the ecological distribution or prevalence, of these high-quality non-redundant genomes in the intestinal microbiota of adults. For this purpose, a total of 4,020 publicly available Illumina shotgun metagenomics-based fecal samples of healthy adults, covering 81 different cohorts from various geographical areas, were selected, and used for such a strain tracking exercise (Supplementary Table S1). Subsequently, strain prevalence and average ANI data were integrated into a specific index score, the AxP, as previously described (Fontana et al., 2022). This analysis revealed that among the selected publicly available genomes, *B. longum* subsp. *longum* strain GCA_015551245.1 showed the highest AxP score, thus highlighting the latter as the ecologically and genomically most representative reference strain for the *B. longum* subsp. *longum* species (Supplementary Table S4). However, since this strain is not publicly available, all genomes of *B. longum* subsp. *longum* strains from our in-house microbial repository were screened to identify the closest related strain to the above-defined optimal model microorganism by using the previously assessed RefBifSelector, which is a tool specifically established for biobank screening based on genomic and functional similarity to an identified model strain (Fontana et al., 2022). This screening assay revealed that the highest score was obtained for *B. longum* subsp. *longum* PRL2022, indicating this strain as a very closely related strain to the above identified reference strain for the *B. longum* subsp. *longum* species (Supplementary Table S5). However, to validate this *in silico*-driven strain selection, genomic insights and *in vitro* analyses were performed to assess PRL2022 safety as well as its ability to cope with human gastrointestinal challenges and to interact with both host cells and other intestinal microorganisms (see also Supplementary text for further information).

### Unique genomic features of PRL2022 with respect to other *Bifidobacterium longum* subsp. *longum* probiotic strains

To assess unique features encoded by the PRL2022 genome that may for example enhance its fitness or gut-associated microbe-host/microbe-microbe interactions, a comparative genomics analysis was performed by considering the genomes of 10 *B. longum* subsp. *longum* strains currently included in commercially probiotic products (Tarracchini et al., 2022). Specifically, to provide a fully representative PRL2022 genome sequence, the PRL2022 genome generated by combining sequencing data obtained from both Illumina sequencing (sequencing output of 556,485 short reads) and Oxford Nanopore MinION (sequencing output of 57,198 long reads) was used. This analysis revealed the presence of 279 genes in the PRL2022 genome that are not present in the genomes of other probiotic *B. longum* subsp. *longum* strains. Insights into these genes highlighted that PRL2022 is provided with “unique genes” involved in the breakdown of complex polysaccharides (Supplementary Table S6), including, genes encoding a β-galactosidase and α-L-arabinofuranosidase (GH39 and GH43) (Supplementary Table S6). These enzymes are required for the degradation of a complex polysaccharide commonly present in plant cell walls, i.e., arabinogalactan, whose degradation has been demonstrated to be strain-dependent within the taxon *Bifidobacterium longum* subsp. *longum* (Fujita et al., 2019; Wang and LaPointe, 2020; Sasaki et al., 2022). Furthermore, PRL2022 was shown to possess various genes involved in the transport/uptake of glycans as well as various glycosyl transferases (Supplementary Table S6). These findings suggest that PRL2022 can activate specific (poly)saccharide-related degradative and transport strategies to compete with other intestinal microorganisms for nutrients. In addition, various genetic sequences corresponding to proteins or protein domains involved in adhesion to/interaction with human intestinal epithelial cells, including Ig-like domain, fibronectin type III domain, von Willebrand-domain, and collagen-binding surface protein, were identified as unique genes in PRL2022 (Supplementary Table S6). This indicates that PRL2022 possesses specific genetic features to facilitate interaction with intestinal cells to ensure its colonization and persistence in the human intestine. Finally, the PRL2022 genome harbors “unique genes” that may have a role in limiting the growth of other microorganisms, including various cysteine/histidine-dependent aminohydrolases/peptidases (CHAP) domain-containing protein as well as a type II toxin-antitoxin system (Supplementary Table S6). Specifically, while CHAP domain-containing proteins exhibit lytic activity toward peptidoglycans (Guglielmetti et al., 2014), the toxin-antitoxin system is involved in bacterial persistence, biofilm formation, and antibiotic tolerance, with the toxin causing cell death or suppression of cell proliferation and antitoxin acting as an antagonist of the toxin action (Klimina et al., 2019, 2020). Therefore, PRL2022 appears to possess specific strategies to not only adhere to/interact with the host and compete for nutrients, but also to limit growth of other microorganisms ensuring its colonization and survival in the competitive human intestinal environment.

### Evaluation of the ability of PRL2022 strain to interact/communicate with the human host thorough *in vitro* assays

**Figure 1:**
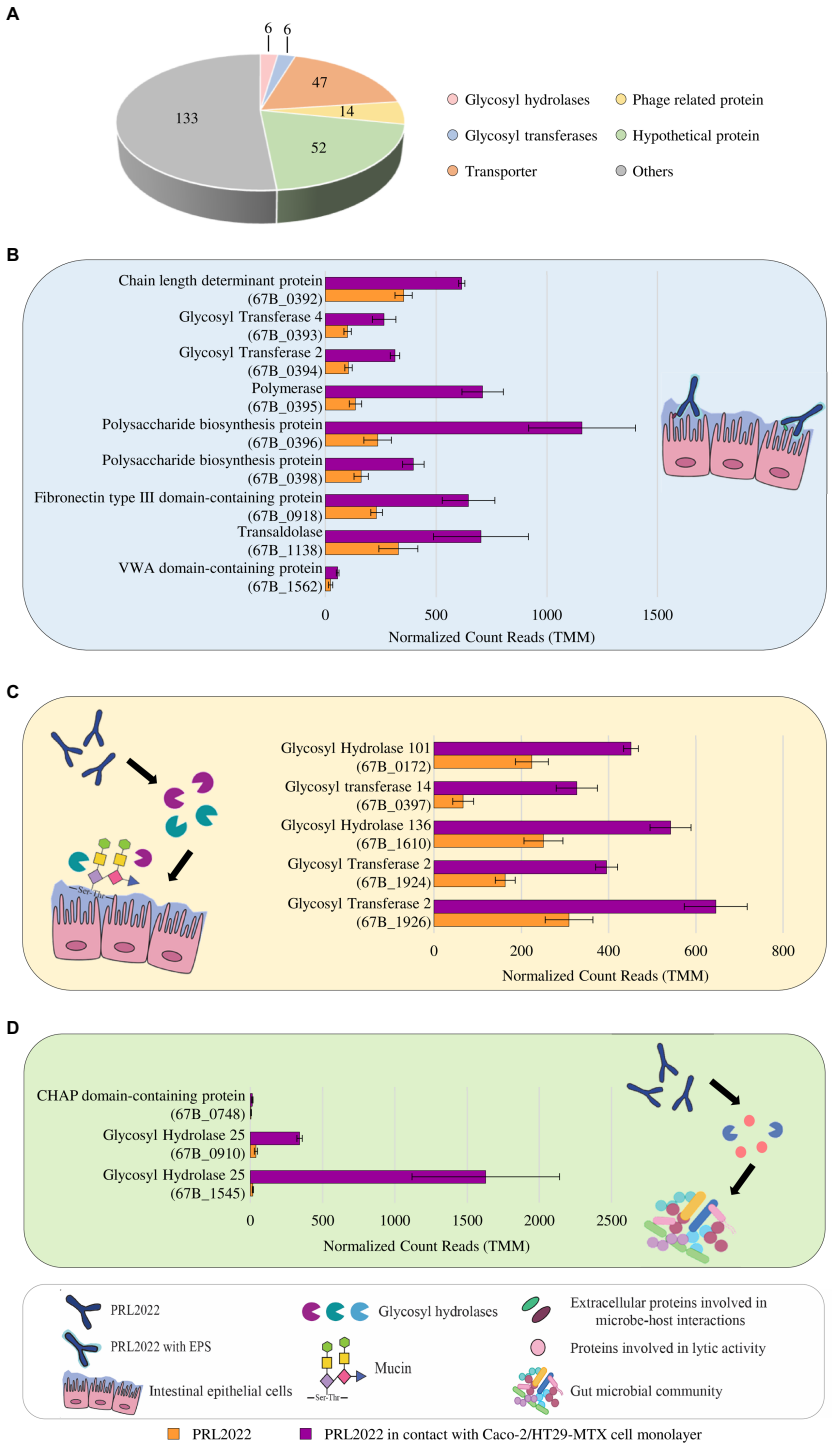
Statistically significant gene expression modulation of *B. longum* subsp. *longum* PRL2022 after 4 h of contact with Caco-2/HT29-MTX monolayer. Panel **(A)** summarizes the number of genes per functional category among the transcriptionally up-regulated genes of PRL2022. The image reports transcriptional modulation of genes, expressed as average of the normalized count reads obtained from each independent biological triplicate, involved in adhesion to the intestinal epithelium [panel **(B)**], mucin degradation and uptake of the derived saccharides [panel **(C)**], and cell-to-cell communications [panel **(D)**]. Each bar plot shows the average of the normalized count reads obtained.

A key biological feature that may be exerted by a bacterium belonging to the autochthonous human gut microbiota, such as PRL2022, is represented by its ability to establish cross-talk with the host intestinal epithelium. Therefore, to investigate possible strategies with which PRL2022 may interact with the host, thereby promoting its fitness and survival in the human intestinal environment, *B. longum* subsp. *longum* PRL2022 was seeded on a Caco-2/HT29-MTX cell monolayer for 4 h. Subsequently, changes in gene expression between PRL2022 cells in contact with the human cell line and those not exposed to this human cell monolayer, i.e., bacterial control cells, were evaluated through RNA sequencing, as previously described (Fontana et al., 2022), generating a total of 7,653,473 quality-filtered reads with an average of 1,275,579 reads per sample. In this context, only genes showing a fold-change of ≥2 in combination with a *value of p* ≤0.05 calculated through correction for multiple comparisons using the False Discovery Rate (FDR) procedure were considered as significantly differentially expressed between the two conditions. Specifically, a total of 449 genes were found to be differentially expressed by comparing the transcriptome of PRL2022 following contact with human cell lines to that of the control (Supplementary Table S7). Interestingly, in depth functional scrutiny of 258 PRL2022 genes whose transcription was up-regulated upon being in contact with human cell lines revealed the presence of a gene encoding a O-antigen ligase (67B_0395), three genes encoding polysaccharide biosynthesis/chain length determinant proteins (67B_0392, 67B_0396, 67B_0398), and two genes coding for glycosyltransferases (GT2 and GT4) (67B_0393 and 67B_0394), all predicted to belong to the genetic locus of PRL2022 genome responsible for exopolysaccharide (EPS) production (Figure 1; Supplementary Table S7). In this context, since EPS have been described as complex glycans exposed on bacterial cell surface directly involved in promoting both bacterial adhesion to the epithelium and microbe-host interactions (Alessandri et al., 2019, 2021), it can be postulated that PRL2022 enhances EPS production as a strategy to adhere to and interact with the human intestinal epithelial cells. In a similar manner, when PRL2022 was placed in contact with the human cell line monolayer, specific transcriptional induction of particular genes occurred, corresponding to proteins implied in the establishment of a microbe-host interaction, i.e., genes encoding a transaldolase (67B_1138) as well as a fibronectin type III domain-containing protein (67B_0918) and a von Willebrand domain-containing protein (67B_1562) (Figure 1; Supplementary Table S7). Specifically, although only demonstrated under *in vitro* experimental conditions, bifidobacterial transaldolases have been described to act as extracellular appendages promoting adherence to the host mucosal surface, therefore acting as adhesive moonlighting proteins with high binding affinities to mucin (Nishiyama et al., 2020; Shang et al., 2022). Proteins harbouring a fibronectin type III- or von Willebrand domain have been shown to promote anchoring to the extracellular matrix protein (Heidler et al., 2021; Nezametdinova et al., 2021; van Leeuwen et al., 2021; Webster et al., 2022). These results indicate that PRL2022 can employ different strategies, including strain-specific features, to adhere to the human intestinal epithelium and, therefore, persist in the human intestinal environment.

Furthermore, beyond genes predicted to be involved in host adhesion, PRL2022 cells exposed to human cell lines showed several upregulated genes predicted to be responsible for the degradation of host-derived glycan secreted by the HT29-MTX cells, i.e., mucin, and for the uptake of the resulting oligosaccharides (Figure 1). Specifically, the transcriptome of PRL2022 exposed to Caco-2/HT29-MTX cell monolayer showed a statistically significant transcriptional upregulation of genes encoding GH101, acting as an endo-α-N-acetylgalactosaminidase, GH136 specifically identified as an extracellular lacto-N-biosidase but also capable of degrading mucin glycan core structure, as previously described (Yamada et al., 2017), as well as GT2, and GT14 both involved in transfer of mucin-related saccharide building blocks, i.e., N-acetylgalactosamine and N-acetylglucosamine (Figure 1). In this context, since mucin is a host-derived carbon source accessible only to a limited number of bacterial strains colonizing the human gut (Tailford et al., 2015), these findings suggest that, when exposed to a human intestine-simulating environment, PRL2022 activates specific carbohydrate degradation strategies to ensure its survival and persistence in the extremely competitive intestinal environment.

Finally, exposure to human cell lines caused a statistically significant higher transcription of PRL2022 genes involved in bacterial cell-to-cell communication, including two genes (67B_0910 and 67B_1545) predicted to encode lysozyme (GH25) and an above-identified unique gene (67B_0748) whose translation produces a cysteine/histidine-dependent aminohydrolases/peptidases (CHAP) domain-containing protein (Figure 1). Interestingly, similar proteins have been shown to exhibit lytic activity toward peptidoglycans (Guglielmetti et al., 2014), suggesting that PRL2022 not only induces mucin degradation genes as a strategy to survive in the competitive intestinal environment, but also produces enzymes potentially able to actively limit growth of other microorganisms.

Altogether, these observations not only suggest the ability of PRL2022 to interact with the host to ensure its survival and persistence in the intestinal microbial ecosystem, but also the multifactorial nature of this process involving microbial surface components and specific carbohydrate-degrading enzymes.

### Assessment of host-activated genes in response to PRL2022 cell exposure

**Figure 2:**
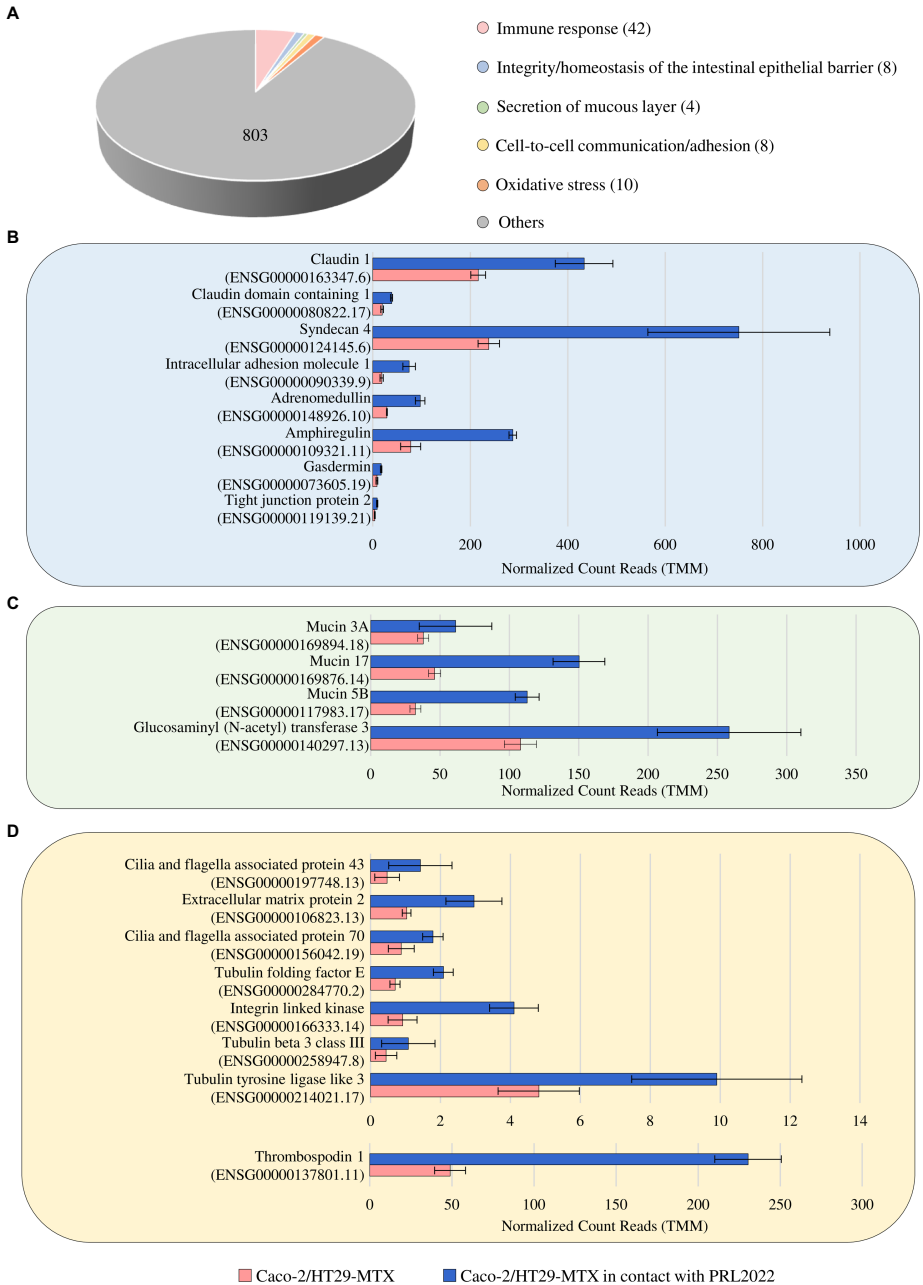
Statistically significant up-regulated genes of Caco-2/HT29-MTX cell monolayer after 4 h of contact with PRL2022. Panel **(A)** reports the number of genes per functional category among the up-regulated genes of the human cell lines when in contact with PRL2022. Panels **(B–D)** display, respectively, transcriptional modulation of those genes involved in integrity/homeostasis of the intestinal epithelial barrier, secretion of mucous layer, and cell-to-cell communication/adhesion. Transcriptional modulation of genes was reported as average of the normalized count reads obtained from each independent biological triplicate.

To investigate if and how the contact between PRL2022 and the Caco-2/HT29-MTX cell monolayer causes modulation of gene expression in the eukaryotic cells, RNA extracted from the human cell line following exposure to PRL2022 was subjected to RNA sequencing, generating a total of 133,961,309 reads with an average of 22,326,885 reads per sample. As described above, only genes showing a fold-change of ≥2 and a *value of p* ≤0.05 calculated through the FDR correction for multiple comparisons procedure were considered as significantly differentially expressed in the Caco-2/HT29-MTX cells in the presence and absence (control samples) of the selected bifidobacterial strain. Interestingly, 875 out of the 1,254 genes differentially expressed in the two conditions were shown to be upregulated in the eukaryotic cells exposed to PRL2022 when compared to the control (Supplementary Table S8). Among these, a gene responsible for the production of a glucosaminyl (N-acetyl) transferase that catalyzes the formation of core 2 and 4 O-glycans on mucin-type glycoprotein, together with three genes, i.e., MUC3A, MUC17, and MUC5B, encoding two mucin-related epithelial glycoproteins and for one of the major gel-forming mucin protein, respectively, were identified (Figure 2). These findings suggest that PRL2022, perhaps because of its ability to utilize mucin, acts as a stimulus for eukaryotic cells to produce/secrete components of the mucous layer covering the human intestinal epithelium, potentially contributing to the reinforcement of the intestinal barrier integrity. Furthermore, various other host genes such as those encoding for the tight junction protein, claudin-1, syndecan, adrenomedullin, amphiregulin, and gasdermin directly or indirectly involved in preserving the integrity/homeostasis of the intestinal epithelial barrier, were significantly more expressed in the human cell monolayer exposed to PRL2022 when compared to the unexposed control (Supplementary Table S8; Bode et al., 2008; Martinez-Herrero and Martinez, 2016; Chen et al., 2018; Frohling et al., 2018; Nishii et al., 2020; Hindson, 2022). These observations reinforce the notion that PRL2022 plays a role in fortifying the intestinal barrier and its ability to stimulate health-promoting effects to the host.

In addition to the genes responsible for maintaining epithelial barrier integrity, various host genes encoding extracellular matrix proteins as well as proteins associated to cilia formation showed a significantly higher transcription level in Caco-2/HT29-MTX cells following PRL2022 exposure (Figure 2). Remarkably, these proteins are known to mediate cell-to-cell communication/adhesion favoring the establishment of an intimate dialog between prokaryotic and eukaryotic cells (Milani et al., 2017; Jeffery, 2019; Vaca et al., 2020), indicating that the presence of PRL2022 induces the production of host proteins involved in host–microbe interaction/adhesion ensuring its persistence in a human intestine-simulating environment. At the same time, a plethora of host genes encoding pattern recognition receptors and cell signaling molecules both involved in eliciting an immune response by the host were shown to be significantly upregulated in eukaryotic cells when placed in contact with PRL2022 cells (Supplementary Table S8). Interestingly, while several host genes associated with pro-inflammatory protein/molecule production were induced in the cell monolayer as a natural result of exposure to bifidobacterial cells, genes coding for anti-inflammatory molecules were also over-expressed. This suggests that PRL2022, as observed for several other bifidobacterial strains, can engage in cross-communication with the host immune system, yet without causing a detrimental inflammatory cascade response, but rather alerting the immune system to promptly react to the possible presence of pathogens (Fanning et al., 2012; Turroni et al., 2013; Longhi et al., 2020).

Overall, these observations suggest that PRL2022 can induce not only the production of extracellular proteins by human intestinal epithelial cells to favor host cell adhesion and, therefore, its persistence in the intestinal environment, but also the overexpression of genes involved in guaranteeing intestinal barrier integrity and priming the immune system.

### Disentangling the molecular interaction of PRL2022 cells with other human gut microorganisms

**Figure 3:**
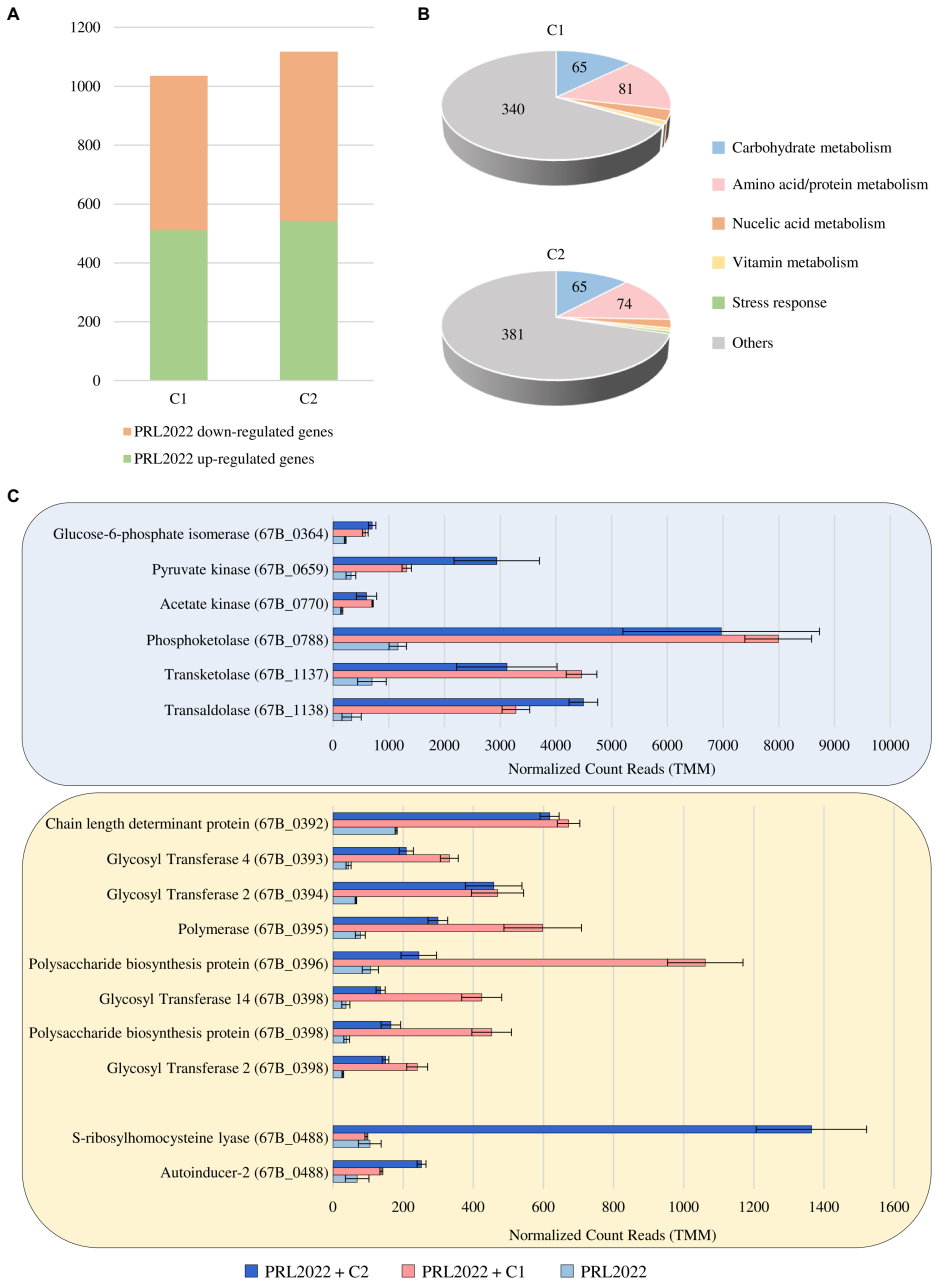
Transcriptional modulation of PRL2022 genes when exposed to either of two stabilized intestinal bacterial communities C1 and C2. Panel **(A)** reports the number of up-regulated and down-regulated genes in PRL2022 when exposed to C1 and C2 when compared to the control. Panel **(B)** displays the number of genes per functional category among the up-regulated genes of PRL2022 in the presence of C1 or C2. Panel **(C)** shows the transcriptional modulation of genes predicted to be involved in acetate and EPS production as well as in quorum sensing-based cell-to-cell communication. Transcriptional modulation of genes was reported as average of the normalized count reads obtained from each independent biological triplicate.

To assess possible cross-talk of PRL2022 cells with other bacterial players of the human intestinal microbiota, co-cultivation assays of this strain with two synthetic microbial communities previously stabilized through a bioreactor-based fermentation system were performed to generate an environment that mimics the human intestine (Alessandri et al., 2022). Specifically, the two synthetic microbial communities were selected to allow co-cultivation of PRL2022 in the presence of a high abundance of species of the genus *Bacteroides* (C1) or *Prevotella* (C2) to mimic an intestinal environment typical of the two most widespread enterotypes of the adult human gut microbiota (Arumugam et al., 2011; Costea et al., 2018). To identify PRL2022 genes whose transcription is modulated following 8 h of co-cultivation, RNA extracted from co-cultures as well as from PRL2022 cells grown under the same conditions yet not exposed to other microbial players was sequenced. RNA sequencing generated a total of 31,369,940 quality-filtered reads with an average of 3,485,549 reads per sample. Specifically, only genes with a fold-change of ≥2 coupled with a *value of p* ≤0.05 based on FDR correction were considered as differentially expressed between PRL2022 cells in the presence or absence of the C1 or C2 microbial ecosystem. In detail, of the 1,035 and 1,117 genes that were determined to be significantly differentially transcribed, a total of 511 and 543 genes were shown to be significantly up-regulated in PRL2022 cells when co-cultivated with either C1 or C2 microbial communities, respectively (Figure 3; Supplementary Table S9). Interestingly, most of the PRL2022 genes showing increased transcription when co-cultivated with the C1 microbial community (compared to the PRL2022 culture without co-cultivation) were shown to correspond to PRL2022 genes that also exhibited increased transcription in the presence of the C2 microbial ecosystem, suggesting that PRL2022 adopts similar response strategies to human intestinal microbiota exposure regardless of the taxonomic composition of the latter. Particularly, PRL2022 cells were shown to increase transcription of a wide variety of genes involved in the metabolism and transport of amino acids/proteins and carbohydrates when co-cultivated with other intestinal microbes (Figure 3; Supplementary Table S9). Probably, the presence of other microorganisms may not only have stimulated PRL2022 to activate multiple strategies to compete for nutrients, but it may also have made accessible, through cross-feeding or resource-sharing activities, specific nutrients that PRL2022 alone would not be able to use (Bunesova et al., 2018; Turroni et al., 2018b). Directly linked to the higher abundance of over-expressed genes involved in carbohydrate degradation, the interaction with other microorganisms combined with the exposure to an intestinal-simulating environment, seem to favor also the increased transcription of PRL2022 genes involved in acetate production, i.e., glucose-6-phosphate isomerase, pyruvate kinase, acetate kinase, phosphoketolase, transketolase, and transaldolase (Figure 3; Supplementary Table S9; Lanigan et al., 2019). These observations emphasize the important interactomic features of this *B. longum* subsp. *longum* strain with the human host microbiota. Indeed, since acetate can be consumed by certain members of the gut microbiota to generate butyrate, a short chain fatty acid known for its beneficial activity to the host promoting intestinal barrier integrity and anti-inflammatory response, the enhancement of the expression of genes involved in the production of acetate by PRL2022 may have an *in vivo* butyrogenic effect and, consequently, exerting health-promoting effects upon the host (Riviere et al., 2016; Alessandri et al., 2019).

Furthermore, and as also observed when PRL2022 interacts with a Caco-2/HT29-MTX cell line monolayer, when exposed to a human intestinal-simulating environment with the presence of a microbial community, the selected strain was shown significantly increase transcription of eight genes of the PRL2022 *eps* locus, specifying four GTs, the polymerase, and three genes coding for polysaccharide biosynthesis protein/chain length determinant protein (Supplementary Table S9; Figure 3). This result indicates that (increased) EPS production is the elective strategy adopted by PRL2022 to interact with both the host and other microbial players ensuring its persistence in the human gut.

Finally, although PRL2022 displayed a similar behavior in response to the interaction with two different microbial communities, only the exposure to C2 induced in the selected bifidobacterial strain a significantly higher number of transcripts of genes predicted to encode S-ribosylhomocysteine lyase (LuxS), i.e., the enzyme responsible for production of autoinducer-2 (AI-2) (67B_0488), and autoinducer-2 ABC transporter (67B_1938) (Supplementary Table S9; Figure 3). These two proteins are involved in production and transport of AI-2, allowing an intricate cell-to-cell communication system, known as quorum sensing, which is able to regulate diverse phenomena, including virulence, adherence to the host cells or biofilm formation (Sun et al., 2014). This suggests that PRL2022 activates this specific interspecies communication system particularly in presence of certain bacterial species.

Overall, these observations highlight the ability of PRL2022 to activate multiple strategies to survive and persist in the competitive gut environment and to interact with other intestinal microbial players.

## Conclusion

*B. longum* subsp. *longum* strains are highly prevalent in the human gut and several strains of this species have been associated with exerting health-promoting effects upon their host. In this context, to identify a *B. longum* subsp. *longum* strain that could act as a valuable model for high fitness in the human intestine, an ecological and phylogenomic approach was applied. This approach allowed the identification of *B. longum* subsp. *longum* PRL2022 as a strain that is, from a genetic and functional point of view, a very close relative of the most representative strains of this bifidobacterial species in the human intestinal microbiota. In-depth insights into PRL2022 genomic determinants coupled with a comparative genome analysis involving PRL2022 and genomes of those *B. longum* subsp. *longum* strains earmarked as model microorganisms of this species and included in commercially available probiotic products revealed the presence of a plethora of genes, some of which identified as PRL2022 unique genes, involved in host- and microbe-microbe interactions. This indicates that PRL2022 possesses a genetic makeup that could favor the survival, colonization, and persistence of this strain in the competitive human intestinal ecological niche. A notion that was corroborated through functional genomic experiments using *in vitro* models mimicking the human intestinal environment and microbial biodiversity, highlighting the ability of PRL2022 to establish extensive cross-talk with both the host and other intestinal microbial players. Therefore, the proposed *in silico* method based on ecological- and phylogenomic-driven selection of a potential *B. longum* subsp. *longum* model strain candidate with a presumed high fitness in the human gut should be considered as a valuable tool to be applied to other (bifido) bacterial species to identify model strains among the autochthonous strains of the human gut.

## Data availability statement

The data presented in the study are doposited in the NCBI SRA repository, accession number PRJNA914637 and PRJNA833139. Furthermore, the updated genome sequence of B. longum subsp. longum PRL2022 is available under the accession number PRJNA692178.

## Author contributions

GA wrote the manuscript and performed *in vitro* analyses. SR, MB, GT, MC, and CA performed *in vitro* analysis. GA, FF, and CT performed bioinformatics analyses. GAL and LM validated the bioinformatic analyses. GL, RA, and AV performed the sequencing. CM, FT, OB, and DS supervised the project and edited the manuscript. MV supervised the project and designed the study. All authors contributed to the article and approved the submitted version.

## Funding

The PhD fellowship of CA is financially supported by Fondazione Cariparma, Parma, Italy.

## Conflict of interest

FF, GL, RA, and AV were employed by GenProbio Srl.

The remaining authors declare that the research was conducted in the absence of any commercial or financial relationship that could be construed as potential conflict of interest.

## Publisher’s note

All claims expressed in this article are solely those of the authors and do not necessarily represent those of their affiliated organizations, or those of the publisher, the editors and the reviewers. Any product that may be evaluated in this article, or claim that may be made by its manufacturer, is not guaranteed or endorsed by the publisher.

## Footnotes

^1^https://github.com/broadinstitute/strainge

^2^https://github.com/rrwick/Filtlong

^3^https://github.com/ExpressionAnalysis/ea-utils
