# Supplementary material for: Identification of a prototype human gut Bifidobacterium longum subsp. longum strain based on comparative and functional genomic approaches
Source: Front Microbiol. 2023 Feb 8;14:1130592. doi: 10.3389/fmicb.2023.1130592 (PMC9945282; doi:10.3389/fmicb.2023.1130592)
Supplement: Supplementary file 2 [file Data_Sheet_1.docx]

**Supplementary Text**

**Insights into PRL2022 genomic determinants predicted to be involved in host- and microbe-microbe interactions.** To evaluate the possible presence of genomic traits in *B. longum* subsp. *longum* PRL2022 conferring biological features predicted to be involved in its interaction with the host and other intestinal microbial players, gene prediction was performed using the MEGAnnotator pipeline (Lugli et al., 2016). In this context, since the catabolism of complex dietary carbohydrates by specific enzymes, i.e., glycosyl hydrolases (GH), enhances digestion by degrading nutrients that would otherwise not be digested by the host and ultimately provides health benefits to the host by releasing, via fermentation, health-promoting compounds, including short chain fatty acids, the PRL2022 glycobiome, i.e., the metabolic enzyme arsenal involved in polysaccharide breakdown, was investigated (Turroni et al., 2018;Alessandri et al., 2021;Tarracchini et al., 2022). Based on the CAZy database, a total of 63 genes were predicted to encode various GHs, encompassing β-L-arabinofuranosidase, α-amylase, xylanase, endo-inulinase, and α- and β-glucosidase, suggesting the potential ability of this strain to degrade a broad range of diet-derived complex glycans. However, beyond the presence of GHs responsible for diet-derived polysaccharide degradation, the PRL2022 genome also possessed certain GHs putatively involved in host-derived glycan catabolism, such as endo-α-N-acetylgalactosaminidase (GH101) and α-N-acetylgalactosaminidase (GH27 and GH129) as well as lacto-N-biosidase (GH20). Furthermore, 47 genes of PRL2022 were predicted to encode various glycosyl transferases (GTs), while nine and four genes correspond to carbohydrate-binding modules and carbohydrate esterases, respectively (Table S10). Thus, the predicted carbohydrate utilization features of PRL2022 suggest an expanded ability of this strain to survive in the human gut and colonize the competitive environment of the human intestine by activating multiple strategies to access nutrients.

Furthermore, bifidobacteria are known to produce different extracellular structures localized on the cell surface, including pili, exopolysaccharides (EPS), and teichoic acids, that promote adhesion to the intestinal epithelium and, thus, drive microbe-host dialogue/interaction (Alessandri et al., 2021;Tarracchini et al., 2022). In this context, functional annotation of PRL2022 genes highlighted the presence of a cluster of genes predicted to be responsible for EPS production, encompassing a pGTF, essential for initiation of EPS subunit biosynthesis, and additional genes corresponding to ABC transporters, subunit polymerization enzymes, and various carbohydrate precursor biosynthesis/modification enzymes (Table S10). In addition, the genome of PRL2022 was shown to encompass gene clusters for teichoic acid and pilus biosynthesis (Table S10).

Furthermore, scrutiny of the PRL2022 genome highlighted the presence of two genes coding for a bile salt hydrolase and a serpin (serine protease inhibitor) (Table S10), which are expected to support the strain in surviving human gastrointestinal challenges, providing tolerance to the deleterious exposure to bile salts and protection against host-derived proteolytic action, respectively (Alvarez-Martin et al., 2012;Ruiz et al., 2012a;Ruiz et al., 2012b;Alessandri et al., 2019).

Overall, PRL2022 genomic traits highlighted the presence of a genetic equipment that could potentially favor the survival, colonization and persistence of this strain in the extremely competitive gut environment.

***In vitro* evaluation of tolerance of *B. longum* subsp. *longum* PRL2022 toward human gastrointestinal challenges.** To reach, colonize, and persist in their preferred ecological niche, i.e., the large intestine, bifidobacterial strains have to counter the adverse and hostile environmental conditions of the upper compartments of the human gastrointestinal tract (GIT), including exposure to bile acids, osmotic stress, or reduced pH (Bottacini et al., 2014;Alessandri et al., 2021). Therefore, a key feature of a potential model gut microorganism is to cope with these challenging gastrointestinal conditions. In this context, to test the ability of PRL2022 to survive osmotic insults or the presence of bile salts, the strain was exposed to high concentrations of NaCl (2%, 6%, and 10%) and oxgall (0.5%, 1%, and 2%) for 3h, after which cell viability was monitored through flow cytometry. Interestingly, with a survival rate > 93% for all the three tested NaCl concentrations, PRL2022 appeared to considerably tolerate osmotic stress (Table S11). At the same time, PRL2022 exposure to oxgall revealed a viability reduction of about 50% for the highest concentration tested, while a survival rate of 88.56% and 70.79% was recorded for 0.5% and 1% of oxgall, respectively (Table S11). These findings suggest that PRL2022 is able to withstand the *in vivo* presence of biliary salts, since the physiological concentration of these compounds in the human GIT has been estimated to be on average 0.4% w/v (Russell et al., 2011). In addition to NaCl and bile salt, the ability of PRL2022 to survive in acidic environment was also evaluated. Interestingly, while PRL2022 incubation for 2h showed a survival rate of 94.79% at pH 4.0, significant reduction in viability with a survival rate of 15.45% and 16.28% was obtained at pH 2.0 and pH 3.0, respectively (Table S11). However, despite the drastic reduction of PRL2022 viability when the strain was exposed to harsh acidic conditions, a survival rate of > 10% indicates that starting from the initial inoculum of ~10^8^ cells/ml, at least 10^7^ cells/ml can survive at extremely acid pH. Furthermore, the survival rate observed at pH 2.0 and 3.0 did not differ from those recorded for other model gut candidate bifidobacterial strains (Serafini et al., 2013;Achi and Halami, 2019;Yasmin et al., 2020). These results validate PRL2022’s highly efficient (adapt)ability to withstand stress from an acidic environment.

Overall, *in vitro* monitoring of PRL2022 survival in some of the stressful conditions that gut microbial strains may encounter on their way along the GIT indicates that this strain has a high tolerance to hostile environments typical of parts of the human GIT.

***B. longum* subsp. *longum* PRL2022 susceptibility to antibiotics.** Beyond their ability to survive extreme environmental conditions, human gut prototype strains ideally should not carry antibiotic resistance genes, since these may be horizontally transferred to other (pathogen) intestinal microorganisms (Kim et al., 2018;Yasmin et al., 2020). In this context, EFSA has defined Minimum Inhibitory Concentration (MIC) breakpoint values for a panel of eight antibiotics above which bifidobacterial strains should not show growth to be considered as potential feed additive (EFSA, 2012). Therefore, MIC assays were performed for the eight antimicrobial compounds suggested by EFSA for PRL2022 coupled with *B. longum* subsp. *longum* ATCC 15707^T^ considered as a quality control strain (ES ISO 10932:2010), revealing that PRL2022 MIC breakpoints were equal to or lower than the established EFSA cut-off values for all tested antibiotics, thus underscoring the safety, in terms of antibiotic resistance phenotype, of this *B.* *longum* subsp. *longum* strain (Table S12).

**References**

Achi, S.C., and Halami, P.M. (2019). In Vitro Comparative Analysis of Probiotic and Functional Attributes of Indigenous Isolates of Bifidobacteria. *Curr Microbiol* 76**,** 304-311.

Alessandri, G., Ossiprandi, M.C., Macsharry, J., Van Sinderen, D., and Ventura, M. (2019). Bifidobacterial Dialogue With Its Human Host and Consequent Modulation of the Immune System. *Front Immunol* 10**,** 2348.

Alessandri, G., Van Sinderen, D., and Ventura, M. (2021). The genus bifidobacterium: From genomics to functionality of an important component of the mammalian gut microbiota running title: Bifidobacterial adaptation to and interaction with the host. *Comput Struct Biotechnol J* 19**,** 1472-1487.

Alvarez-Martin, P., O'connell Motherway, M., Turroni, F., Foroni, E., Ventura, M., and Van Sinderen, D. (2012). A two-component regulatory system controls autoregulated serpin expression in Bifidobacterium breve UCC2003. *Appl Environ Microbiol* 78**,** 7032-7041.

Bottacini, F., Ventura, M., Van Sinderen, D., and O'connell Motherway, M. (2014). Diversity, ecology and intestinal function of bifidobacteria. *Microb Cell Fact* 13 Suppl 1**,** S4.

Efsa (2012). Guidance on the assessment of bacterial susceptibility to antimicrobials of human and veterinary importance. *EFSA Journal*.

Kim, M.J., Ku, S., Kim, S.Y., Lee, H.H., Jin, H., Kang, S., Li, R., Johnston, T.V., Park, M.S., and Ji, G.E. (2018). Safety Evaluations of Bifidobacterium bifidum BGN4 and Bifidobacterium longum BORI. *Int J Mol Sci* 19.

Lugli, G.A., Milani, C., Mancabelli, L., Van Sinderen, D., and Ventura, M. (2016). MEGAnnotator: a user-friendly pipeline for microbial genomes assembly and annotation. *FEMS Microbiol Lett* 363.

Ruiz, L., O'connell-Motherway, M., Zomer, A., De Los Reyes-Gavilan, C.G., Margolles, A., and Van Sinderen, D. (2012a). A bile-inducible membrane protein mediates bifidobacterial bile resistance. *Microb Biotechnol* 5**,** 523-535.

Ruiz, L., Zomer, A., O'connell-Motherway, M., Van Sinderen, D., and Margolles, A. (2012b). Discovering novel bile protection systems in Bifidobacterium breve UCC2003 through functional genomics. *Appl Environ Microbiol* 78**,** 1123-1131.

Russell, D.A., Ross, R.P., Fitzgerald, G.F., and Stanton, C. (2011). Metabolic activities and probiotic potential of bifidobacteria. *Int J Food Microbiol* 149**,** 88-105.

Serafini, F., Strati, F., Ruas-Madiedo, P., Turroni, F., Foroni, E., Duranti, S., Milano, F., Perotti, A., Viappiani, A., Guglielmetti, S., Buschini, A., Margolles, A., Van Sinderen, D., and Ventura, M. (2013). Evaluation of adhesion properties and antibacterial activities of the infant gut commensal Bifidobacterium bifidum PRL2010. *Anaerobe* 21**,** 9-17.

Tarracchini, C., Viglioli, M., Lugli, G.A., Mancabelli, L., Fontana, F., Alessandri, G., Turroni, F., Ventura, M., and Milani, C. (2022). The Integrated Probiotic Database: a genomic compendium of bifidobacterial health-promoting strains. *Microbiome Res Rep*.

Turroni, F., Milani, C., Duranti, S., Mahony, J., Van Sinderen, D., and Ventura, M. (2018). Glycan Utilization and Cross-Feeding Activities by Bifidobacteria. *Trends Microbiol* 26**,** 339-350.

Yasmin, I., Saeed, M., Khan, W.A., Khaliq, A., Chughtai, M.F.J., Iqbal, R., Tehseen, S., Naz, S., Liaqat, A., Mehmood, T., Ahsan, S., and Tanweer, S. (2020). In vitro Probiotic Potential and Safety Evaluation (Hemolytic, Cytotoxic Activity) of Bifidobacterium Strains Isolated from Raw Camel Milk. *Microorganisms* 8.
